# Investigating the Ischaemic Phase of Skin NADH Fluorescence Dynamics in Recently Diagnosed Primary Hypertension: A Time Series Analysis

**DOI:** 10.3390/jcm12041247

**Published:** 2023-02-04

**Authors:** Regina Pawlak-Chomicka, Wojciech Chomicki, Tomasz Krauze, Paweł Uruski, Maria Guzik, Jarosław Piskorski, Andrzej Tykarski, Przemysław Guzik

**Affiliations:** 1 Department of Hypertensiology, Angiology and Internal Medicine, Poznan University of Medical Sciences, 61-848 Poznan, Poland; 2 Department of Physics of Functional Materials, Faculty of Physics, Adam Mickiewicz University, 61-614 Poznan, Poland; 3 Department of Cardiology-Intensive Therapy and Internal Medicine, Poznan University of Medical Sciences, 60-355 Poznan, Poland; 4 Faculty of Biology, Medicine and Health Sciences, University of Manchester, Manchester M13 9PL, UK; 5 Institute of Physics, University of Zielona Gora, 65-516 Zielona Gora, Poland

**Keywords:** arterial hypertension, 460-nm skin fluoresce, mitochondria, ischaemia, nicotinamide adenine dinucleotide, flow-mediated skin fluorescence

## Abstract

The reduced form of nicotinamide adenine dinucleotide (NADH) is crucial in cellular metabolism. During hypoxia, NADH accumulation results from anaerobic cytoplasmic glycolysis and impaired mitochondrial function. This study aimed to compare the dynamic changes in the 460-nm forearm skin fluorescence, which reflects cellular NADH content, during transient ischaemia between healthy individuals and patients with newly diagnosed, untreated essential hypertension (HA). Sixteen healthy volunteers and sixty-five patients with HA underwent non-invasive measurement of forearm skin NADH content using the Flow Mediated Skin Fluorescence (FMSF) method at rest and during a 100-s transient ischaemia induced by inflation of the brachial cuff. The fluorescent signal was sampled at 25 Hz. All samples were normalised to the end of the ischaemic phase, which is the most stable phase of the whole recording. Slope values of 1 s linear regressions were determined for every 25-sample neighbouring set. The 1-s slopes in the early phase of skin ischaemia, indicating quicker hypoxia-induced NADH accumulation in skin, were significantly higher in patients with HA than in healthy individuals. These findings suggest that some protecting mechanisms postponing the early consequences of early cellular hypoxia and premature NADH accumulation during skin ischaemia are impaired in patients with untreated HA. Further studies are needed to investigate this phenomenon.

## 1. Introduction

Arterial hypertension (HA) is a complex disease with multiple contributing factors. Endothelial dysfunction and microcirculation damage have been linked to HA [1,2]. In recent years, mitochondrial dysfunction has also been identified as a potential contributor to HA development. Improving mitochondrial function has been suggested to be beneficial in managing this disease [3].

Mitochondria are critical to various metabolic pathways and intracellular signalling networks, playing a key role in producing adenosine triphosphate (ATP) and many biosynthetic intermediates. Their dysfunction is associated with various diseases (e.g., muscular dystrophy or Alzheimer’s disease) [4,5]. 

The reduced form of nicotinamide adenine dinucleotide (NADH) is essential for cellular metabolism. Under normal oxygen conditions, NADH is processed in the mitochondrial electron transport chain (ETC) to generate ATP [6]. However, in hypoxia, NADH is not oxidised to nicotinamide adenine dinucleotide (NAD+), leading to a cellular accumulation of NADH [7]. 

The Flow Mediated Skin Fluorescence (FMSF) is a non-invasive method for measuring NADH fluorescence in the skin during transient ischaemia and reperfusion [8]. FMSF measures the 460-nm skin fluorescence of the forearm at rest and during transient, controlled ischaemia caused by external compression of the brachial artery via brachial cuff inflation. After deflation of the cuff, blood flow returns, initiating post-ischaemic reperfusion. 

During the no-flow phase through the brachial artery, oxygen is not supplied to the forearm, leading to hypoxemia/anoxia metabolism typical of ischaemia. At this time, the skin’s 460-nm fluorescence gradually increases from the resting baseline. During reperfusion, blood flow and oxygen supply are restored, and the fluorescence rapidly drops below the resting baseline. After a few minutes, the fluorescence recovers to baseline values [9].

FMSF reflects dynamic mitochondrial function related to blood flow through skin microcirculation [9,10,11]. It has been used to study healthy individuals and athletes at rest and during various challenges (exercise, long-term training, repeated ischaemia and reperfusion to initiate preconditioning). FMSF has also been studied in patients with coronary artery disease [12], diabetes [13], obstructive pulmonary disease [14], and lupus [8,10,15]. Resting cellular NADH increases with age and in several cardiovascular diseases [9,13], obstructive pulmonary disease [14], and diabetes [13]. We have recently shown that skin 460-nm fluorescence increases in patients with HA after a 6-week treatment with metoprolol. The treatment with amlodipine, perindopril, or nebivolol did not affect this fluorescence [11]. Studying the skin’s fluorescence during no-flow ischaemia provides a unique opportunity to examine the effects of hypoxia on mitochondria and cells without the confounding factor of blood flow. By focusing on the gradual accumulation of NADH, it is possible to gain insight into the cellular and mitochondrial response to hypoxia since its initiation. However, the dynamic response of cells and mitochondria to hypoxia in individuals with HA has not been previously studied.

This study aimed to compare the skin’s NADH fluorescence during transient ischaemia between healthy individuals and patients with newly diagnosed and untreated primary HA. Our primary intention was to better understand the cellular and mitochondrial response to hypoxia in individuals with primary HA using a non-invasive in vivo model of transient skin ischaemia.

## 2. Materials and Methods

Sixty-five patients with newly diagnosed primary HA were enrolled. Primary HA was diagnosed based on 24 h ambulatory blood pressure monitoring (ABPM) according to the European Society of Hypertension 2018 Guidelines [1]. The following blood pressure criteria were used to diagnose AH: ≥ 130/80 mmHg over 24 h or ≥135/85 mmHg for the daytime average or ≥120/70 for the night time average.

Two clinical inclusion criteria were used for patient enrolment: (1) newly diagnosed and previously untreated primary HA with systolic blood pressure (SBP) <180 and/or diastolic blood pressure (DBP) <110 mmHg, and (2) sinus rhythm on an electrocardiogram. Exclusion criteria included secondary HA, other chronic diseases such as diabetes, chronic obstructive pulmonary disease, cancer, heart failure, atrial fibrillation, renal impairment (with estimated glomerular filtration rate <60 mL/min/1.73 m^2^), acute inflammation, pregnancy or breastfeeding, or regular use of any drugs. Various diagnostic tests were conducted to rule out the possibility of secondary HA from common aetiologies. Among them were routine biochemical analysis, resting electrocardiography, transthoracic echocardiography, and, if necessary, endocrine function assessments and renal artery ultrasonography. Additionally, 16 healthy volunteers were recruited for the control group.

### 2.1. Flow Mediated Skin Fluorescence

A detailed description of the FMSF method can be found in previous publications [8,16]. In brief, FMSF continuously and non-invasively measures the 460-nm fluorescent light emitted by the skin in response to earlier excitation by the 340-nm ultraviolet light. The ultraviolet light does not reach deeper than 0.5 mm from the skin surface, so the FMSF method provides information from the most superficial skin cells [12,17,18].

For FMSF, all measurements were acquired from the forearm skin at rest and then during short-term controlled ischaemia evoked by the complete closure of the brachial artery by the blood pressure cuff and during reperfusion. The results are presented as a fluorescence curve. In this study, we limited our analysis of the FMSF curve to the rest and ischaemic phase only.

### 2.2. The Ischaemic Phase of the 460 nm Forearm Skin Fluorescence

Before the FMSF test, each participant’s blood pressure was measured to set a target value of 60 mmHg over SBP for the brachial cuff inflation. Blood pressure was measured in a sitting position, with a standard cuff on the upper limb resting on a tabletop, after 5 min of rest in silence. In order to determine the blood pressure value, the average of three consecutive readings, taken at one-minute intervals, was calculated [1]. An operator manually entered the determined pressure value into the device. It is a prerequisite for starting the FMSF measurement, which then happens automatically. After the rest phase, the cuff was inflated to a predetermined target pressure and held for 100 s to squeeze the brachial artery and stop the blood flow transiently. The inflation of the brachial cuff is fully automatic and usually takes less than 3 s to reach the target air pressure squeezing the artery. The deflation stopping the ischaemia phase is faster and ends in approx. 1 s.

The end of brachial artery occlusion, corresponding to the moment of cuff deflation at peak ischaemia (PI), was taken as the reference point for each participant. There are two reasons for this choice. First, it is the most stable phase of the entire recording after the 460-nm skin fluorescence reaches the plateau. Second, during the ischaemia phase, there is no blood flow, and microcirculation does not affect the measured signal at this time.

All samples of the 460-nm fluorescence signal were normalised to the reference value, i.e., divided and presented as its fraction. At rest, many oscillations are visible in the recorded and normalised signals. They are secondary to the pulsatory nature of blood flow through the skin microcirculation. For our analysis, we took the last 20 s of the resting baseline, followed by the 100 s of ischaemia.

**Figure 1:**
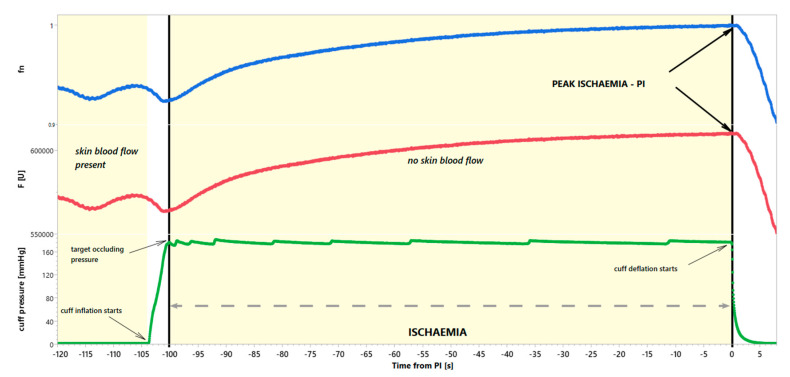
Example of NADH fluorescence plot after normalization to peak ischaemia (blue) and the corresponding fragment of the NADH florescence before normalisation (red) with visualisation of pressure changes in the cuff occluding the brachial artery (green). Fn—fluorescence after normalisation to PI, F[u]—fluorescence in absolute units.

**Figure 2:**
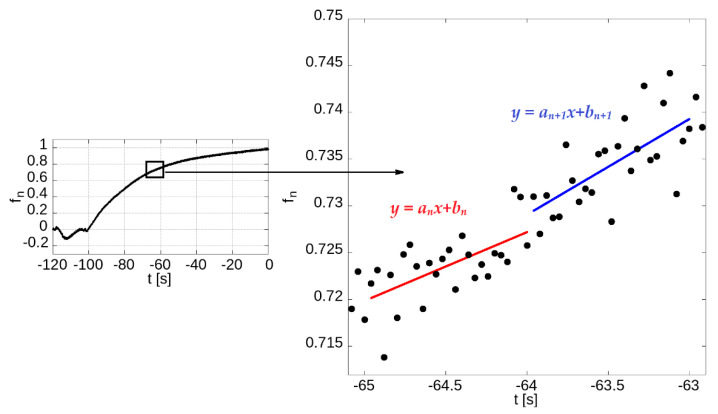
An example of the NADH fluorescence trace with a schematic view of the method for determining the slope coefficients *a* of the line (where the line equation is described by the Formula *y = ax + b*) for points in neighbouring 1-s intervals. The diagram shows two examples of lines with the Equations *y = a_n_x + b_n_* and *y = a_n+1_x + b_n+1_*.

Each skin fluorescence sample was synchronised and then normalised to the PI’s fluorescence. Figure 1 illustrates all mathematical operations. The data prepared in this way were quantified using simple linear regression determined for 25 consecutive measurements of 460-nm normalised fluorescence using the least squares method. Consecutive 25 samples cover exactly 1-s segments of the FMSF signal. Regression lines drawn over consecutive time series values reflect the average dynamics of an increase or reduction in a specific signal. Positive slopes correspond to increasing trends, while negative slopes correspond to declining trends. Slopes close to 0 indicate no dynamics in the analysed time series segment. The values of the slope of line a (where the equation of the line is described by the Formula y = ax + b) were calculated for points in successive consecutive time intervals lasting 1 s (Figure 2).

### 2.3. Estimation of Sample Size

As there are no data for the normalised FMSF signal, we could not refer to any known studies or reference values. Therefore, we used data from our previous study on the 6-week effects of primary hypertension treatment with different antihypertensive drugs on the FMSF. Although the minimal estimated sample size for that study was 14, we investigated more patients.

### 2.4. Statistical Analysis

The Shapiro–Wilk test of clinical data and slopes showed that the distribution of the continuous data was not normal; thus, all data are summarised as median and the 25th and 75th percentiles (Q1–Q3). As a result, the nonparametric Mann–Whitney test was used to compare patients with primary hypertension and healthy individuals. Differences with a *p*-value <0.05 were considered statistically significant. All statistical analyses were performed using Statistica (StatSoft, Poland, version 13).

### 2.5. Ethics Approval Statement

The study was designed and conducted following the principles outlined in the Declaration of Helsinki. The Bioethical Committee at Poznan University of Medical Science approved the study protocol and all forms (approval no. 435/17, Annex 243/19). Written informed consent was obtained from all participants.

## 3. Results

Women made up 38% of the HA patient and control groups. The median age of the patients with HA and healthy individuals was comparable at 40 (Q1–Q3: 34–49) and 38 (Q1–Q3: 30–47) years, respectively (*p* = 0.44). Heart rate during the ABPM was similar between the groups. However, patients with HA had significantly higher SBP and DBP in all ABPM measures than healthy individuals. The mean values of blood pressure and heart rate in ABPM are presented in Table 1.

### Ischaemia-Related Dynamic Changes in the FMSF Signal

**Figure 3:**
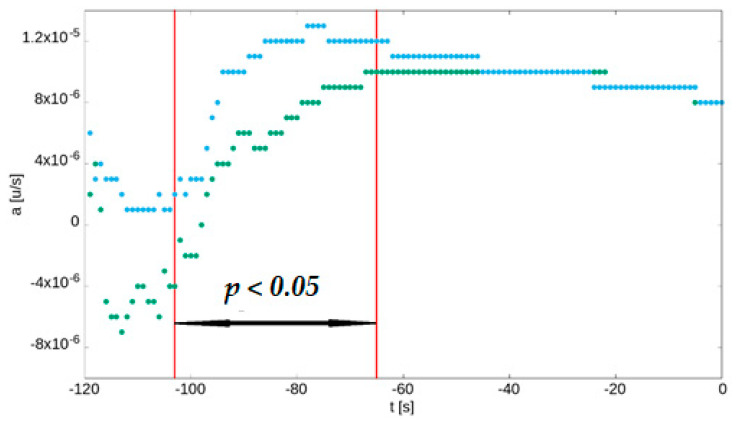
Median values of 1-s slopes (*values of a from linear regression over every 25 consecutive samples of normalised fluorescence*) for rest and the 100-s forearm skin ischaemia. During the earliest skin ischaemia, patients with HA (blue dots) had higher slopes than healthy people (green dots).

The 1-s slopes of linear regressions were significantly higher in patients with HA compared to healthy individuals between 103 and 65 s before the PI. Figure 3 summarises these findings.

## 4. Discussion

We found that patients with primary and untreated HA have a more rapid increase in skin fluorescence compared to healthy individuals during the early stages of skin ischaemia. This phenomenon lasts for over 30 s, suggesting a faster accumulation of NADH in the superficial, visible cells of the skin during ischaemia.

During rest, the skin’s microcirculatory blood flow is preserved. However, after the inflation of brachial cuffs, the flow ceases completely. At this time, all dynamic changes in the 460-nm skin fluorescence cannot be related to the microcirculatory function but to intracellular processes involving NADH. The NADH accumulation appears to start before the target +60 mmHg over SBP is achieved in the brachial cuff. It is likely due to the gradual, although very fast, inflation of the brachial cuff. The effects of brachial artery squeezing on the artery lumen can be seen as the participant’s DBP is overcome. Then, the gradual onset of cellular hypoxia in the superficial skin cells starts. This process appears very fast, and the 460-nm skin fluorescence is very sensitive, detecting the first change event before the target blood pressure of 60+ mmHg over SBP is reached.

Microcirculatory and endothelial dysfunction is present in the pathogenesis of HA. Nevertheless, these processes do not fully explain the different dynamics of NADH growth during ischaemia when blood flow in the microcirculation is arrested. Our findings suggest the involvement of other intracellular mechanisms. 

Arterial wall thickening caused by HA disturbs the exchange of oxygen, nutrients, and metabolites between the intravascular space and surrounding tissues. Consequently, several abnormalities related to chronic or repeated cellular hypoxia may occur [19], such as perivascular inflammation [20] with augmented production of reactive oxygen species, impaired nitric oxide synthesis [21], insulin resistance, and glucose metabolism impairment [22,23,24]. All of these factors modify cellular and mitochondrial functions and energy metabolism. 

In recent years, there has been increasing interest in the relationship between mitochondria and HA. This relationship seems bidirectional.

Increased blood pressure may cause mitochondrial dysfunction and structural adaptation [25]. Hypertension-related anomalies are found in mitochondrial biogenesis, dynamics, mitophagy, structural changes, malfunction in bioenergetics, and antioxidant activity [25,26]. HA damages mitochondria through several mechanisms, including activation of the angiotensin II-mitochondrial inner membrane receptor, an increase in NADH oxidase, mechanical stretch, and extracellular matrix turnover [25]. An impaired mitochondrial function has been involved in HA pathogenesis [26,27]. Dysfunctional mitochondria play a role in HA progression and the development of its complications. 

Substantial overlap exists in the consequences of HA and mitochondrial dysfunction. Impaired mitochondria contribute to vascular ageing, i.e., arterial stiffness, endothelial dysfunction, impaired contractility, vasorelaxation, and target-organs damage [26]. HA causes the same complications. Therefore, mitochondria have become a new therapeutic target in HA treatment [27]. 

Mitochondrial energy production is reduced in HA. It has been associated with a change in substrate use, enzyme defect, sirtuins signal defect, augmented reactive oxygen species (ROS) production, and alterations in mitochondrial dynamics. As a result, cell viability is reduced [26,28]. All of this is linked with increased tissue NADH content [29].

In prehypertensive rats, an increase in ROS production was detected in brain cells [25]. Inflammation and stimulation of angiotensin II promoted the production of ROS [30], likely due to reduced activity of NAD-dependent deacetylase sirtuin 3 [3]. This leads to the deactivation of mitochondrial superoxide dismutase 2 protection against oxidative stress [31]. The increase in ROS production by complex I is associated with a decrease in electron transport chain activity [30], resulting in an increase in NADH/NAD+ and a reduction in ATP content [25].

Regardless of the mechanisms, our findings indirectly show some cellular and mitochondrial dysfunction in patients with untreated hypertension. Notably, this dysfunction is observable in an in vivo non-invasive clinical model with transient ischaemia and in a tissue not typically affected by HA, i.e., skin. 

Recently, we have shown that the FMSF method detected post-ischaemic skin preconditioning after repeated bouts of ischaemia and reperfusion in healthy people [16]. The FMSF also detected a significant increase in mean baseline fluorescence, maximum in ischaemia and minimum in reperfusion after an exercise to exhaustion and after 7-week endurance training in elite athletes [8,15].

### 4.1. Study Limitations

The FMSF method studies skin only over the forearm and not in other areas of the body. In the FMSF, there are three phases preserved (rest), no (ischaemia), and then restored (reperfusion) blood flow. Only during ischaemia are the dynamic changes in the 460-nm skin fluorescence attributed to NADH. During the rest and reperfusion, the fluorescence measurements are strongly influenced by the microcirculatory function and the blood flow. The blood flow effects might be subtracted from the measured fluorescence if the light reflectance was measured [32], but unfortunately, the available Angioexpert device does not have this capability. For this reason, we have studied only the ischaemic part of the FMSF curve to exclude any blood flow effects in skin microcirculation on the 460-nm fluorescence readings.

### 4.2. Perspectives

Transient and controlled ischaemia forces cells to shift into anaerobic metabolism with an increase in lactate and H+ [17,33]. Excessive H+ binds to NAD+ and causes NADH accumulation in mitochondria. The FMSF method, with its ischaemic phase, allows for non-invasive observation of these changes in easily accessible forearm skin [34]. Thus, the FMSF method could serve as a simple, non-invasive method of assessing mitochondrial function in various diseases, including hypertension. This method might also be applied to study the metabolic effects of various medical agents, including those used in HA management [11].

## 5. Conclusions

Compared to the control group, patients with benign-to-moderate and untreated HA present an earlier and more dynamic increase in the ischaemic phase of the FMSF curve. This suggests that NADH accumulates more rapidly during early ischaemia in HA. 

The early ischaemic phase of the FMSF is probably sensitive enough to detect the first signs of mitochondrial metabolic dysfunction in HA. We also demonstrated that the accumulation of NADH during skin ischaemia can be non-invasively studied by the FMSF method. The clinical value of our findings requires further study before they can be validated.

## Figures and Tables

**Table 1:** The mean blood pressure and heart rate values in ABPM in the patients with HA and control group, DBP—diastolic blood pressure, HR—heart rate, Q1–Q3—25th and 75th percentiles. SBP—systolic blood pressure, ST—sleep time, WT—wake time.

	Control Group *n* =16		Patients with HA *n* = 65		*p*-Value (Mann-Whitney Test)
	Median	Q1–Q3	Median	Q1–Q3	
**SBP WT [mmHg]**	129	122–133	148	143–156	<0.01
**DBP WT [mmHg]**	79	76–83	88	84–93	<0.01
**HR WT [beats/min]**	82	77–93	82	76–90	0.96
**SBP ST [mmHg]**	105	102–112	122	113–131	<0.01
**DBP ST [mmHg]**	63	58–69	70	66–77	<0.01
**HR ST [beats/min]**	63	60–69	65	60–73	0.64
**SBP 24-h [mmHg]**	125	117–127	143	138–149	<0.01
**DBP 24-h [mmHg]**	76	71–79	84	81–90	<0.01
**HR 24-h [beats/min]**	77	74–87	79	74–85	0.91
**Baseline fluorescence [kU]**	876	556–1010	687	495–846	0.17
**Peak ischaemic fluorescence [kU]**	966	604–1096	744	536–909	0.17

## Data Availability

Not applicable.
